# Calmodulin binds the N-terminus of the functional amyloid Orb2A inhibiting fibril formation

**DOI:** 10.1371/journal.pone.0259872

**Published:** 2022-01-13

**Authors:** Maria A. Soria, Silvia A. Cervantes, Ansgar B. Siemer

**Affiliations:** Department of Physiology and Neuroscience, Zilkha Neurogenetic Institute, Keck School of Medicine, University of Southern California, Los Angeles, California, United States of America; Russian Academy of Medical Sciences, RUSSIAN FEDERATION

## Abstract

The cytoplasmic polyadenylation element-binding protein Orb2 is a key regulator of long-term memory (LTM) in *Drosophila*. The N-terminus of the Orb2 isoform A is required for LTM and forms cross-β fibrils on its own. However, this N-terminus is not part of the core found in ex vivo fibrils. We previously showed that besides forming cross-β fibrils, the N-terminus of Orb2A binds anionic lipid membranes as an amphipathic helix. Here, we show that the Orb2A N-terminus can similarly interact with calcium activated calmodulin (CaM) and that this interaction prevents fibril formation. Because CaM is a known regulator of LTM, this interaction could potentially explain the regulatory role of Orb2A in LTM.

## Introduction

Cytoplasmic polyadenylation element-binding (CPEB) proteins are important mRNA translational regulators. In *Drosophila melanogaster*, the CPEB homolog Orb2 has been shown to be important for long-term memory (LTM) by regulating mRNA translation at the synapse [1–3]. There are two isoforms of Orb2, Orb2A and Orb2B. Both are present in the synapse, though Orb2B is found abundantly throughout the cytoplasm whereas Orb2A concentrations are low [3]. Although both Orb2A and Orb2B can form cross-β (amyloid) fibrils in vitro, Orb2A is required for Orb2 aggregation in vivo and both isoforms are found together in in vivo aggregates [3–6]. This aggregation is necessary for long-term memory [3]. Much of the work on Orb2 aggregation in memory suggests that Orb2A may be a key regulator of overall Orb2 aggregation [2, 3, 7]. The formation of functional fibrils is thought to be a highly regulated process, in contrast to cross-β fibrils in disease. However, the mechanism that regulates Orb2A aggregation is currently not known.

**Fig 1:**
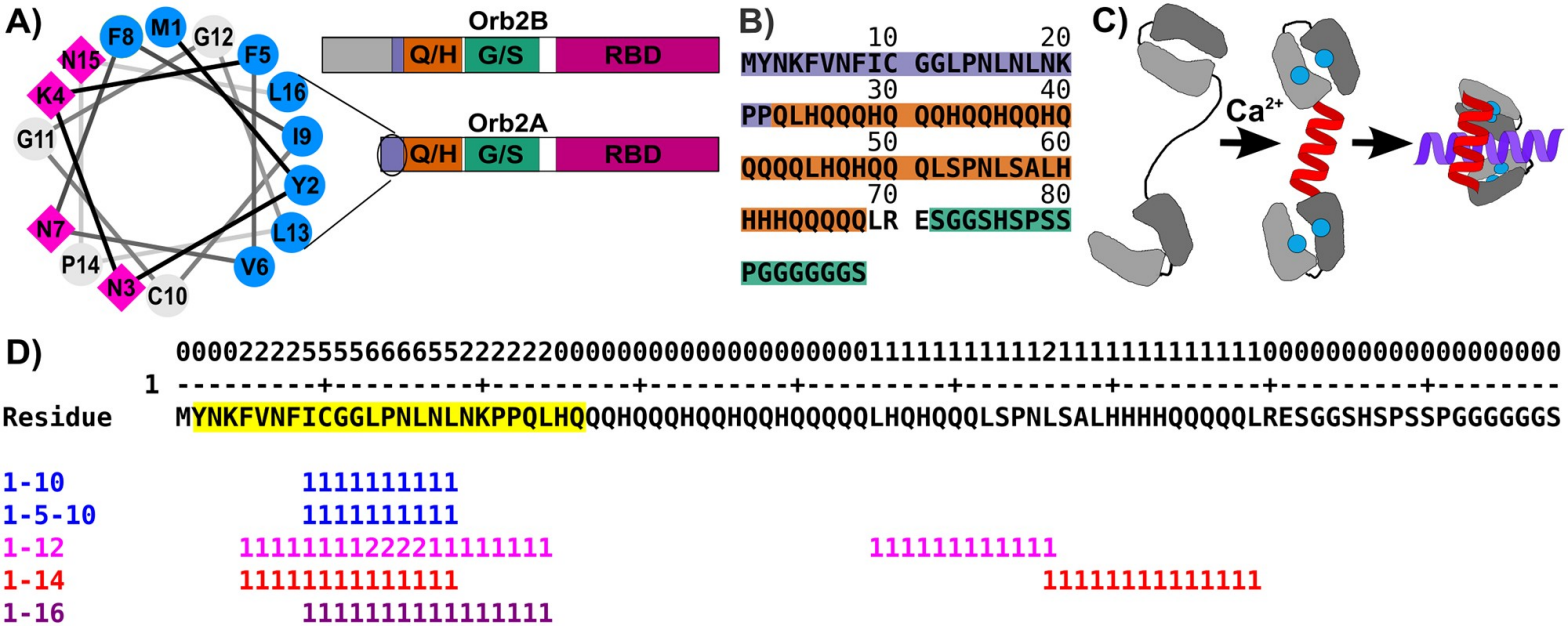
A) Domain layout of Orb2A and Orb2B, including the amphipathic helix at the N-terminus of Orb2A. The extended N-terminus of Orb2B is highlighted in gray, the amphipathic region in purple, the Q/H-rich domain in orange, the G/S-rich domain in green, and the RBD in pink. B) Sequence of the N-terminal 88 amino acids of Orb2A used throughout this study. The colors correspond to the domains in A. C) Mechanism by which Ca^2+^/CaM binds to amphipathic helices. Blue circles represent Ca^2+^, the red helix is the linker domain of CaM, the purple helix is a hypothetical CaM binding partner. D) CaM binding site prediction using the calmodulin meta-analysis predictor. Overall per residue score that is equal to the number of times a residue is found in a canonical CaM-binding motif is shown in the first line followed by the Orb2A_1-88_ sequence with the identified potential binding motif highlighted in yellow. Matches to canonical CaM binding motifs are shown at the bottom.

Orb2A and Orb2B differ only in their N-termini. Orb2A has only 8 N-terminal residues before the first residue shared with Orb2B. The N-terminus of Orb2B is rich in serine and has over 150 residues before the first common residue with Orb2A (Fig 1A). Both Orb2A and Orb2B share a glutamine/histidine-rich (Q/H-rich) domain followed by a glycine-rich domain, two RNA binding domains, and a C-terminal zinc finger. The cryo-EM structure of functional Orb2 fibrils extracted from *Drosophila* brains by Hervás and co-workers showed that the Q/H-rich domain of Orb2 forms the cross-β core [5]. However, the N-terminus of Orb2A, which is not part of this core, was shown to be important for Orb2 fibril formation and LTM: Using random mutagenesis, Majumdar and co-workers showed that the first eight amino acids of Orb2A were over-represented as mutations that inhibited aggregation [3]. Point mutations of residue F5 in Orb2A affected aggregation in vitro and LTM in flies [3, 4]. Using solid-state NMR and electron paramagnetic resonance (EPR), we showed that fibrils formed by the first 88 amino acids of Orb2A had their cross-β core located within these first residues. In fact, the first 21 residues of Orb2A, which precede the Q/H-rich domain, were able to form cross-β fibrils on their own [8]. In addition to being able to form cross-β fibrils, the N-terminal 16 residues of Orb2A form an amphipathic helix when plotted on a helical wheel (see Fig 1A). The combination of an amphipathic helix with a glutamine-rich domain makes Orb2A reminiscent of huntingtin exon-1 (HTTex1) in which an amphipathic N-terminus (N17) precedes a polyQ domain. Based on the fact that the HTTex1 N17 domain was shown to bind lipid membranes [9, 10], we originally investigated the lipid binding capabilities of Orb2A. We showed that the N-terminus of Orb2A forms an amphipathic helix in the presence of anionic lipid vesicles and binds to these vesicles. This binding in turn inhibited the formation of the N-terminal cross-β fibril core [11].

A protein that commonly interacts with amphipathic helices is the calcium-sensing protein calmodulin (CaM). Among its many functions in a large number of different cell types, CaM is an important signal integration protein in neurons for LTM [12]. CaM is present in very high concentrations in the central nervous system, up to 10–100 μM [13], and has been shown to directly regulate several proteins involved in long-term potentiation (LTP). These include adenylyl cyclases AC1 and AC8 [14, 15], calcineurin, and CaM kinases (CaMK) II and IV [16–20]. Depending on the level of Ca^2+^ present, CaM can activate proteins specifically for LTP or long-term depression (LTD) [21], both of which are important in LTM. CaM interacts with sequences that are both amphipathic and positively charged. While there are many modes of binding with CaM depending on the target (reviewed in [22]) the most frequently observed mode relies on the binding of Ca^2+^ to each of the four EF hand domains present in CaM. These four EF hands are separated, two to each side, by an unstructured linker, which becomes α-helical when Ca^2+^ binds to the EF hands (Fig 1C). This creates hydrophobic binding pockets with which many CaM binding partners interact.

In hippocampal dendrites, the CaM/Ca^2+^-regulated kinase CaMKII is responsible for activating mouse CPEB 1 via phosphorylation, which in turn activates mRNA translation [23]. Considering the amphipathic and positively charged nature of Orb2A’s N-terminus, its involvement in LTM, and the high concentration of CaM in the neuron, we decided to investigate whether CaM might directly bind to Orb2A’s N-terminus. Here we show that the N-terminal amphipathic domain of Orb2A indeed binds activated CaM and that this binding affects aggregation of Orb2A_1-88_ into cross-β fibrils.

## Materials and methods

### Protein expression and purification

All Orb2A_1-88_ constructs, cloned into pET28b vectors, were described previously [8]. Wild-type Orb2A_1-88_ (Fig 1B) and cysteine mutants V6C, L18C, and G84C were expressed in *E*. *coli* Rosetta2 (DE3) cells (EMD Millipore), the other Orb2A_1-88_ mutants were expressed using BL21 (DE3) cells. For all protein constructs, the appropriate plasmid was transformed into CaCl_2_ chemically competent cells, and one colony was used to inoculate 25 ml of lysogeny broth (LB) Miller medium with the appropriate antibiotics at 37°C. After approximately 4 h, this culture was diluted into 1 l LB with the appropriate antibiotics and was grown until OD_600_ = 0.6. The culture was then induced with isopropyl β-D-1-thiogalactopyranoside (IPTG), and protein was expressed at 25°C for approximately 16 h. Cell cultures were spun down at 4000 rpm in a Sorvall SLC-6000 rotor (Thermo Fisher Scientific, Waltham, MA) for 20 min at 4°C, and the pellets were stored at -80°C.

To purify protein, cell pellets were thawed and suspended in Denaturing Buffer (8 M urea, 10 mM Tris, 100 mM NaH_2_PO_4_, and 0.05% β-mercaptoethanol (pH 8.0)). The cells were lysed on ice using a Q125 ultrasonic homogenizer (QSonica, Newton, CT). The cell lysate was centrifuged at 20,000 rpm for 20 min using a Sorvall SS-34 rotor. The supernatant was collected and poured onto a Ni-NTA column equilibrated with Denaturing Buffer. The column was then incubated with gentle shaking for ~1 h. For EPR samples, this incubation was skipped. The flowthrough was collected and the column was washed with the following series of solutions: 1) Denaturing Buffer containing 0.5% Triton-X100, 2) Denaturing Buffer containing 500 mM NaCl, 3) Denaturing Buffer at pH 6.75, 4) Renaturing Buffer (200 mM NaCl, 50 mM NaH_2_PO_4_,pH 8.0, 10% glycerol (v/v), and 0.05% (v/v) β-mercaptoethanol), and 5) Renaturing Buffer containing 20 mM imidazole. Orb2A_1-88_ eluted in Renaturing Buffer containing 250 mM imidazole. Samples intended for EPR experiments included an additional washing step: 6) Renaturing Buffer (pH 7.4) without β-mercaptoethanol. EPR samples were also eluted with 250 mM imidazole but at pH 7.4 without β-mercaptoethanol.

### EPR studies

Protein aliquots were thawed on ice, and 2.5 μl of 40 mg/ml S-(1-oxyl-2,2,5,5- tetramethyl-2,5-dihydro-1H-pyr-rol-3-yl) methyl methanesulfonothioate (MTSL) spin label (Toronto Research Chemicals, North York, Ontario, Canada) was added (i.e. in great excess of the protein). The protein-MTSL mixture was incubated at room temperature for 1 h. Then the protein was diluted 15x with deionized water (dH_2_O) and added to a cation exchange column (S Ceramic HyperD F, Pall Life Sciences, Port Washington, NY), which had been equilibrated with dH_2_O. The flow-through was collected and the column was washed with 7 ml dH_2_O. The protein was then eluted with 2 ml of 8 M guanidine-HCl. This was then dialyzed 3x against 1l buffer with 20 mM HEPES, 100 mM NaCl, with or without 10 mM CaCl_2_, depending on if the experiment required Ca^2+^ free conditions or not. Protein aggregates were centrifuged out of solution and the supernatant concentration was measured via its absorption at 280 nm. Finally, the desired amount of CaM, Ca^2+^, EDTA or buffer was added to the protein depending on the experiment, and the sample was loaded into a borosilicate capillary tube (0.6 mm inner diameter, 0.84 mm outer diameter; Vitro-Com, Mt. Lakes, NJ).

Continuous-wave EPR spectra were collected using a Bruker X-band EMX spectrometer (Bruker Biospin, Billerica, MA) at room temperature. For each spectrum 15 scans were accumulated in a high sensitivity cavity at a scan width of 150 gauss. The microwave power was 12.6 mW. Amplitude was calculated as the distance between the highest and lowest points of the spectrum. To determine the dissociation constant, amplitudes at different CaM concentrations were measured in triplicate and fitted to a one-site binding hyperbolic function using the method of least squares using an in house python script based on SciPy and matplotlib [24, 25]. For kinetics curves, readings were taken of the specified sample over the indicated period of time. Amplitudes were normalized to the initial amplitude of each data set, and then plotted together for comparison.

### Thioflavin-T fluorescence assays

Orb2A_1-88_ protein aliquots were thawed on ice and the buffer was changed to 20 mM HEPES, 100 mM NaCl, and, depending on the experiment, 10 mM CaCl_2_ using a PD10 desalting column. In a 96-well plate, Orb2A_1-88_ was mixed with either buffer or CaM at a 1:1 molar ratio to a final concentration of 10 μM. Buffer and CaM by itself were run as controls. ThT was added to each well for a final concentration of 50 μM. Fluorescence kinetics were then acquired at room temperature with gentle periodic mixing in an Eppendorf (Hamburg, Germany) AF2200 plate reader using an excitation wavelength of 440 nm and emission wavelength of 480 nm. Error bars represent the standard deviation of three biological replicates.

### Electron microscopy

Orb2A_1-88_ protein aliquots were thawed, exchanged into 20 mM HEPES, 100 mM NaCl, and the appropriate amounts of Ca^2+^, EDTA, and CaM were added. In addition, 0.02% sodium azide was added to prevent microbial growth. Samples were incubated at room temperature with slight agitation. To image, copper formvar grids (Electron Microscopy Sciences, Hatfield, PA) were incubated with drops of protein sample for 5 min, and then incubated with uranyl acetate (1%) for another 5 min. Grids were then washed with two drops of uranyl acetate and one drop of water and allowed to dry. Finally, grids were imaged using a JEOL JEM-1400 EM (Tokyo, Japan).

## Results

### Prediction of CaM binding sites

We initially applied various bioinformatics tools to see if they predicted the N-terminus of Orb2A to be a CaM binding domain in accordance with our hypothesis. Where the calmodulin target database [26] detected no CaM binding motif, the CaMELS algorithm [27] gave Orb2A_1-88_ an overall interaction score of 0.68, but located the binding site at residue 65 rather than the N-terminus. In contrast, the calmodulin meta-analysis predictor [28] identified a potential CaM binding site at Orb2A residues 2–26, which showed similarities to the canonical 1–10, 1-5-10, 1–12, 1–14, and 1–16 binding motifs (see Fig 1D).

### Orb2A_1-88_ binds to activated CaM

Next, we wanted to test whether Orb2A indeed binds CaM. To do this, we measured EPR spectra of Orb2A_1-88_ 10R1 i.e. spin labeled at its natural cysteine. Residue 10 is located in the amphipathic sequence at the N-terminus of Orb2A which we hypothesized binds to CaM (Fig 1). In addition, using this wild-type cysteine as a reporter leads to minimal perturbation of the protein sequence.

**Fig 2:**
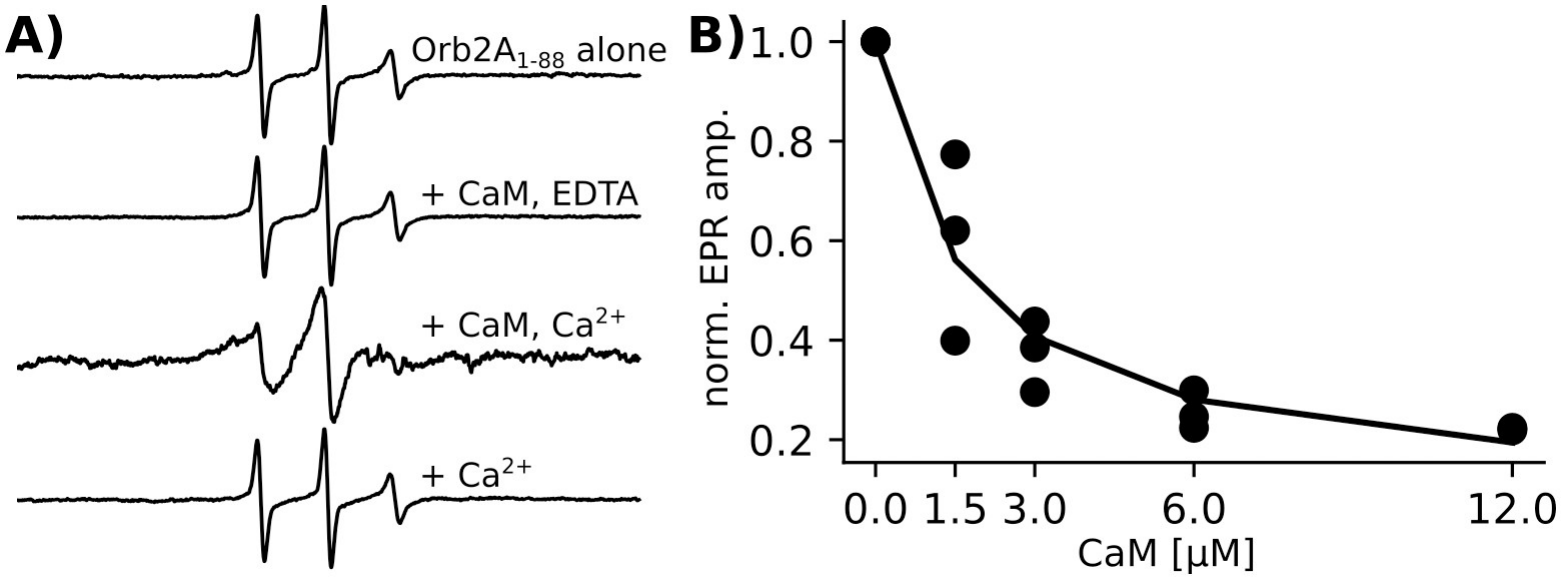
Activated CaM binds Orb2A_1-88_. A) EPR spectra of Orb2A_1-88_ labeled at position 10 (10R1) show no change in linewidth with the addition of CaM together with EDTA or Ca^2+^alone. However, the linewidth increased with the addition of CaM together with Ca^2+^. CaM was added at a 2:1 molar ratio to Orb2A_1-88_, Ca^2+^ and EDTA were added to a final concentration of 1 mM. EPR spectra intensity was normalized to their central linewidth. B) Change in EPR central line amplitude of 6 μM Orb2A_1-88_ 10R1 with increasing concentrations of CaM. Ca^2+^ was present in excess (10 mM). Three biological replicates are shown together with a fit to a one-site binding hyperbolic function to determine the dissociation constant.

After MTSL labeling, we acquired EPR spectra of Orb2A_1-88_ 10R1 in varying conditions: 1) Orb2A_1-88_ 10R1 alone; 2) Orb2A_1-88_ 10R1 in the presence of CaM and EDTA, which assures that CaM is in the inactive conformation; 3) Orb2A_1-88_ 10R1 in the presence of CaM and Ca^2+^, which assures that CaM is in its active conformation; 4) Orb2A_1-88_ 10R1 in the presence of Ca^2+^ as a control. EPR line broadening is an indicator of rigidity at the site of the spin label and frequently used to measure protein binding [29]. As can be seen from Fig 2A, only in the presence of both CaM and Ca^2+^ did we observe line broadening for Orb2A_1-88_ 10R1. Neither CaM in the presence of EDTA nor Ca^2+^ alone caused line broadening suggesting that activated CaM binds the N-terminus of Orb2A.

Line broadening leads to a decrease in amplitude. We therefore used the EPR amplitude to estimate the fraction of Orb2A_1-88_ binding to CaM in a concentration dependent binding curve. This is possible because, unlike linewidth, the EPR amplitude changes linearly with the fraction of bound protein if the unbound and fully bound state have different amplitudes (see supplement of [11]). We again labeled the wild type cysteine 10 with MTSL, and measured EPR spectra at different Orb2A_1-88_ to CaM ratios (Fig 2B). From these data, we were also able to determine a dissociation constant of 1.6±0.5 μM.

### Orb2A_1-88_ binds to CaM within its N-terminal amphipathic domain

**Fig 3:**
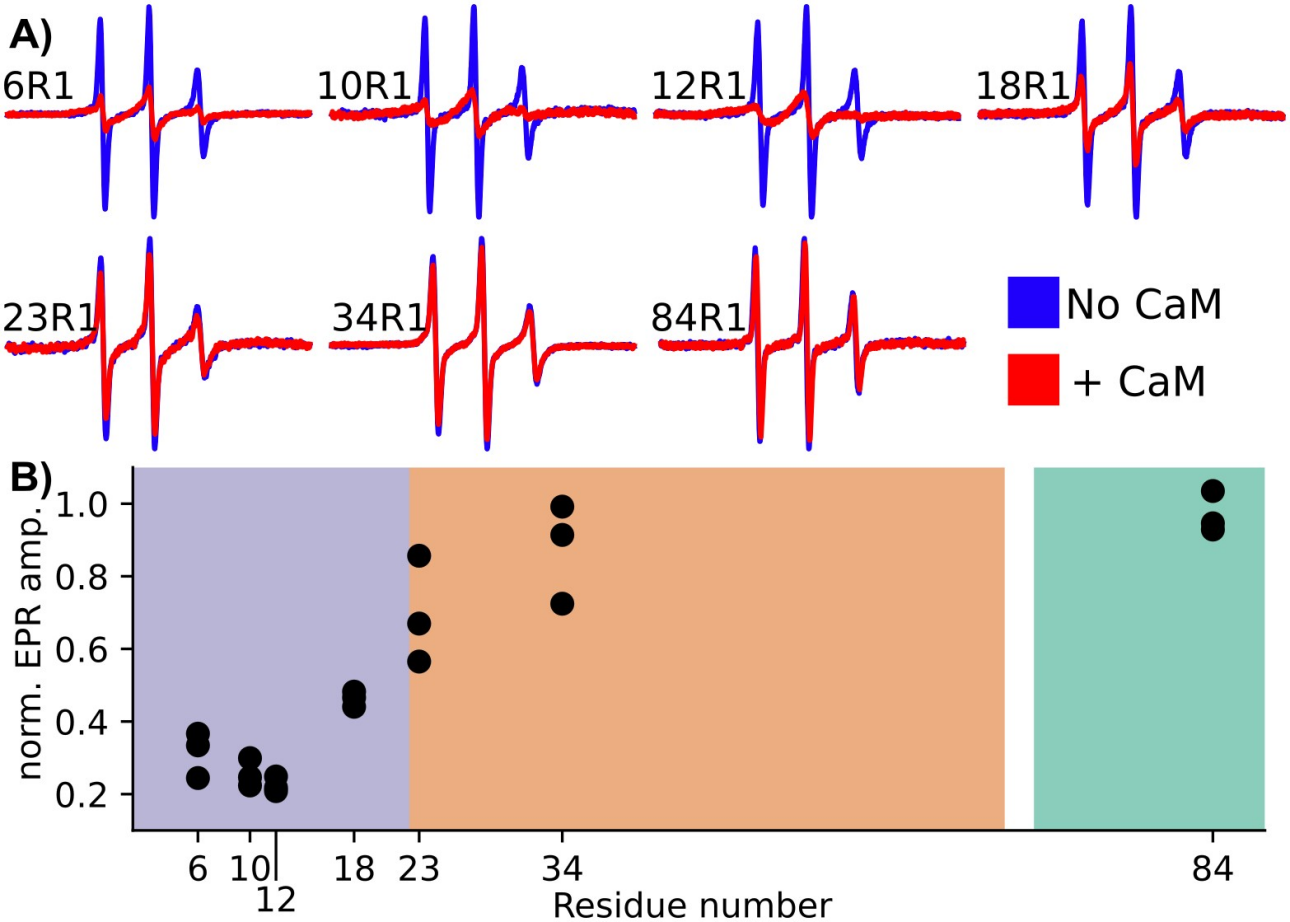
CaM binds the amphipathic N-terminus of Orb2A_1-88_. A) EPR spectra in the absence of CaM (blue) and with the addition of activated CaM (i.e. with 10 mM Ca^2+^) at a 1:1 molar ratio (red). B) Relative EPR amplitude after the addition of CaM compared to without CaM for each site. The low relative amplitude of the EPR spectra for the amphipathic N-terminus (purple) relative to the Q/H-rich domain (orange), and the G/S-rich domain (green), confirms that CaM binds the N-terminus of Orb2A.

While the natural cysteine used for spin labeling in the above experiments was within the N-terminal amphipathic domain, we wanted to get a more specific idea of where on Orb2A_1-88_ CaM was binding. We used cysteine mutants throughout Orb2A_1-88_ for MTSL labeling which we described in previous publications [8, 11]. We acquired EPR spectra in the presence of 10 mM Ca^2+^ both with and without CaM and in a 1:1 molar ratio for each construct. Fig 3A shows the resulting EPR spectra. In the absence of activated CaM the spectra of each site have relatively narrow lines and are quite similar indicating an intrinsically disordered state. The addition of CaM then leads to line boarding predominantly for the N-terminal sites. Fig 3B shows the relative change in amplitude at each site with the addition of CaM. We observed the largest decrease in amplitude at the N-terminus, which confirms that CaM binds the N-terminal amphipathic domain. Spin labels inside the Q/H-rich domain showed little amplitude change and 84R1 at the C-terminus of Orb2A_1-88_ showed no significant change in amplitude in the presence of CaM. These results indicate that neither the Q/H-rich nor the glycine-rich domain are involved in CaM binding.

### CaM binding inhibits aggregation of Orb2A_1-88_

**Fig 4:**
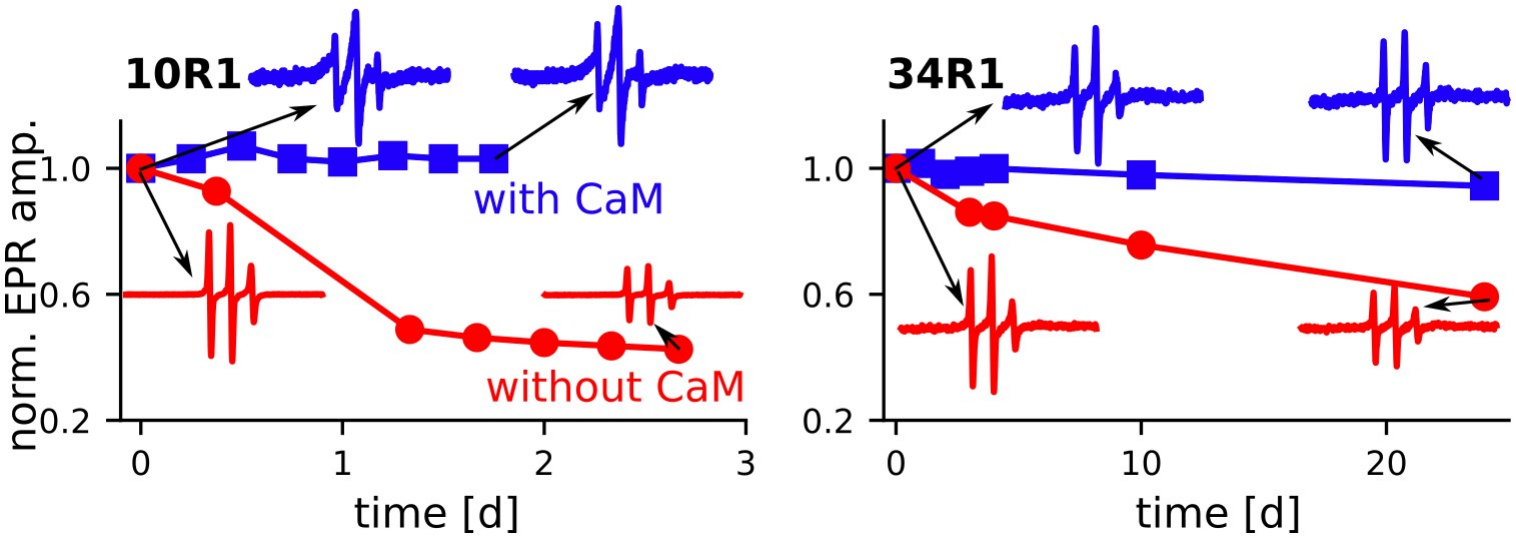
CaM binding prevents Orb2A1-88 aggregation. Change in EPR spectral amplitude of Orb2A_1-88_ labeled at residues 10 and 34 with and without activated CaM (1:2 molar ratio, 10 mM Ca^2+^). Amplitudes were normalized to the first spectrum of each kinetic experiment. In addition, EPR spectra corresponding to the beginning and end of each curve are shown. The EPR amplitude in the absence of CaM decreases significantly for 10R1, i.e. in the center of the amphipathic region, and over time also for 34R1, inside the Q/H-rich domain. This decrease is compatible with aggregation of the protein into cross-β fibrils. No change in amplitude was observed for CaM bound Orb2A_1-88_ suggesting that this interaction prevented fibril formation.

Previously, we showed that the cross-β core of fibrils formed by Orb2A_1-88_ is located within the N-terminal amphipathic domain [8]. Considering that CaM binds to the same domain, we wanted to know whether CaM binding influenced Orb2A_1-88_ fibril formation. We first used EPR to monitor amplitude changes over time in the N-terminal domain at 10R1 (i.e. the natural cysteine). Cross-β fibril formation usually results in a decrease in EPR amplitude [30]. Even though CaM binding already lowers the amplitude, we expect the amplitude to continue to decrease because fibril formation leads to very broad EPR lines at 10R1 due to electron spin-exchange [8]. As can be seen from Fig 4, in the absence of CaM, we observed a decrease in EPR amplitude over two days as expected. However, in the presence of activated CaM, the EPR amplitude did not decrease over time but stayed relatively constant throughout the measurements. This observation indicates that Orb2A_1-88_ bound to CaM is not forming an N-terminal cross-β fibril core.

We then wondered if Orb2A_1-88_ was still forming fibrils but using a different domain as its cross-β core. For example via its Q/H-rich domain that was the core of ex vivo Orb2 fibrils or of the recombinant Orb2ΔRBD fragments that forms fibrils via phase separation [5, 6]. To determine whether or not Orb2A_1-88_ was aggregating in the Q/H-rich domain, we again used EPR to track the change in amplitude over time, but this time the MTLS label was at residue 34 (34R1) i.e. inside the Q/H-rich domain (Fig 4). We observed a slight decrease in amplitude over time for Orb2A_1-88_ without CaM, but we saw no decrease in amplitude in the presence of CaM. This confirms our initial conclusion that without CaM, Orb2A_1-88_ forms fibrils with a cross-β core at its N-terminus, but in the presence of CaM, Orb2A_1-88_ does not aggregate.

### The aggregation inhibited by CaM is cross-β in nature

**Fig 5:**
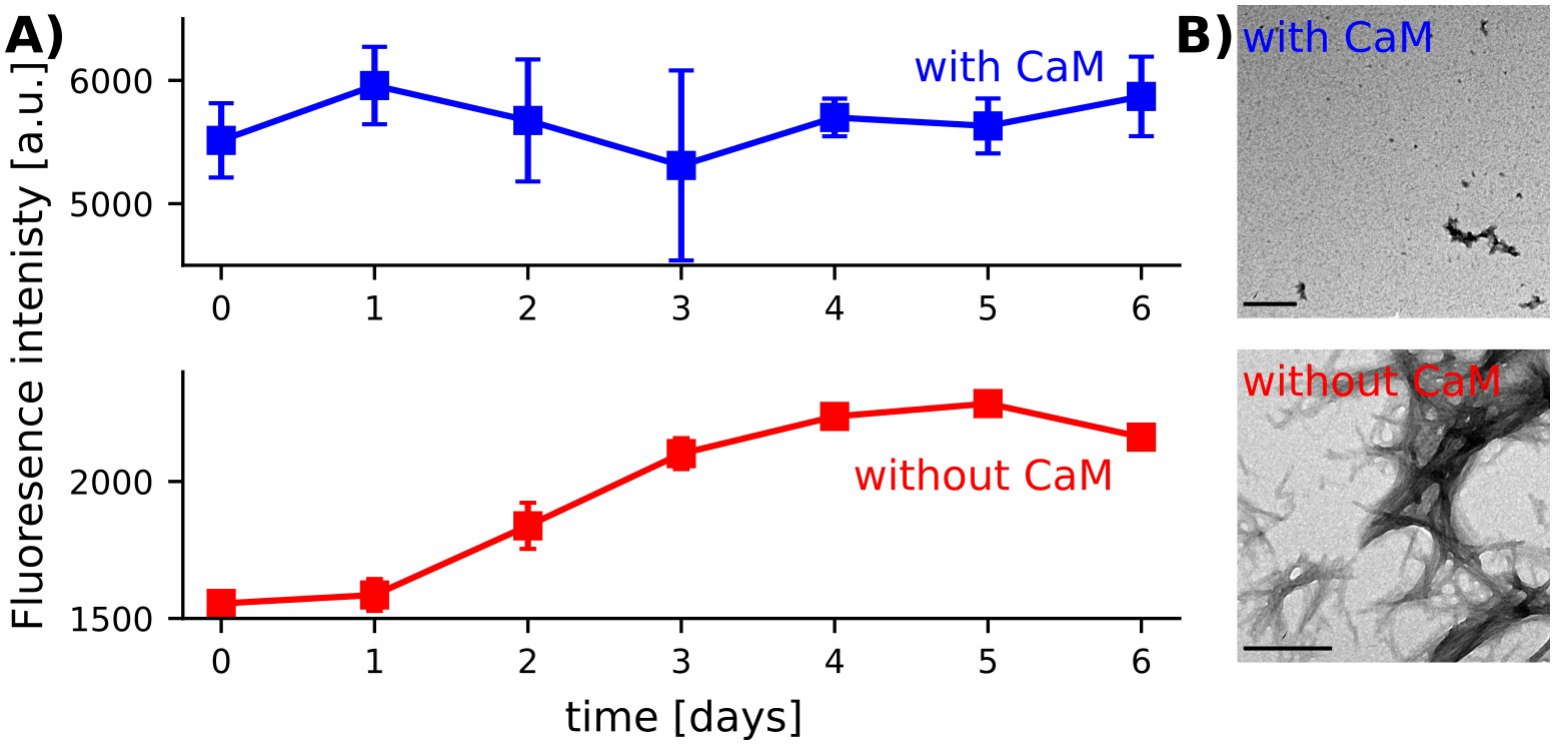
CaM + Ca^2+^ prevents fibril formation of Orb2A_1-88_. A) ThT fluorescence kinetics of Orb2A_1-88_ alone (red) and Orb2A_1-88_ with CaM + Ca^2+^at a 1:1 molar ratio (blue). Error bars representing the standard deviation of three biological replicates are shown in case they are larger than the marker. The fluorescence in the presence of CaM is high because CaM + Ca^2+^ induces Tht fluorescence on its own. B) EM images of samples as described in A, taken after 9 days of incubation.

To confirm that CaM inhibits fibril formation of Orb2A_1-88_, we used Thioflavin-T (ThT) fluorescence, which is commonly used as an indicator of cross-β fibril formation [31]. It is thought that ThT binds to the β-sheet core of a fibril, which stabilizes the rotating bond and causes fluorescence [32, 33]. Because of this, the change in ThT fluorescence can be used to measure fibril forming kinetics. We tracked the ThT fluorescence over time for both Orb2A_1-88_ alone, and Orb2A_1-88_ with CaM (Fig 5A). Orb2A_1-88_ alone gave a characteristic cross-β aggregation curve with a lag phase followed by a sigmoidal increase in fluorescence, and a plateau after about 4–5 days. This increase in Tht fluorescence is somewhat slower than the decrease of EPR amplitude in our 10R1 sample. For Orb2A_1-88_ samples where CaM and Ca^2+^ were present, we did not observe any net change in ThT fluorescence over time. However, the ThT fluorescence of samples with activated CaM was quite high even in the absence of Orb2A_1-88_. We speculate that this is caused by activated CaM binding ThT in its hydrophobic binding pockets.

To confirm these ThT fluorescence results, we used electron microscopy (EM). We saw that while the ThT positive Orb2A_1-88_ sample contained visible fibrils, Orb2A_1-88_ with CaM and Ca^2+^ did not form any structures resembling fibrils (Fig 5B). This supports the conclusion that CaM inhibits cross-β aggregation of Orb2A_1-88_.

## Discussion

In this study, we show that the first 88 amino acids of Orb2A bind to CaM via their N-terminal domain. We previously showed that this domain is able to form an amphipathic helix that is capable of binding anionic lipid membranes, and proposed that Orb2A_1-88_ preferred anionic lipid vesicles because of the lysines present at position 4 and 20, as well as the positively charged N-terminus [11]. Positively charged amphipathic helices have also been shown to be preferred binding partners for CaM [34]. In addition, one CaM binding site prediction algorithm identified the N-terminus of Orb2A as a potential binding site. Our EPR data confirmed this hypothesis showing that calcium activated CaM can bind to the N-terminus of Orb2A_1-88_. In addition, we showed that this interaction prevented the formation of Orb2A_1-88_ fibrils.

**Fig 6:**
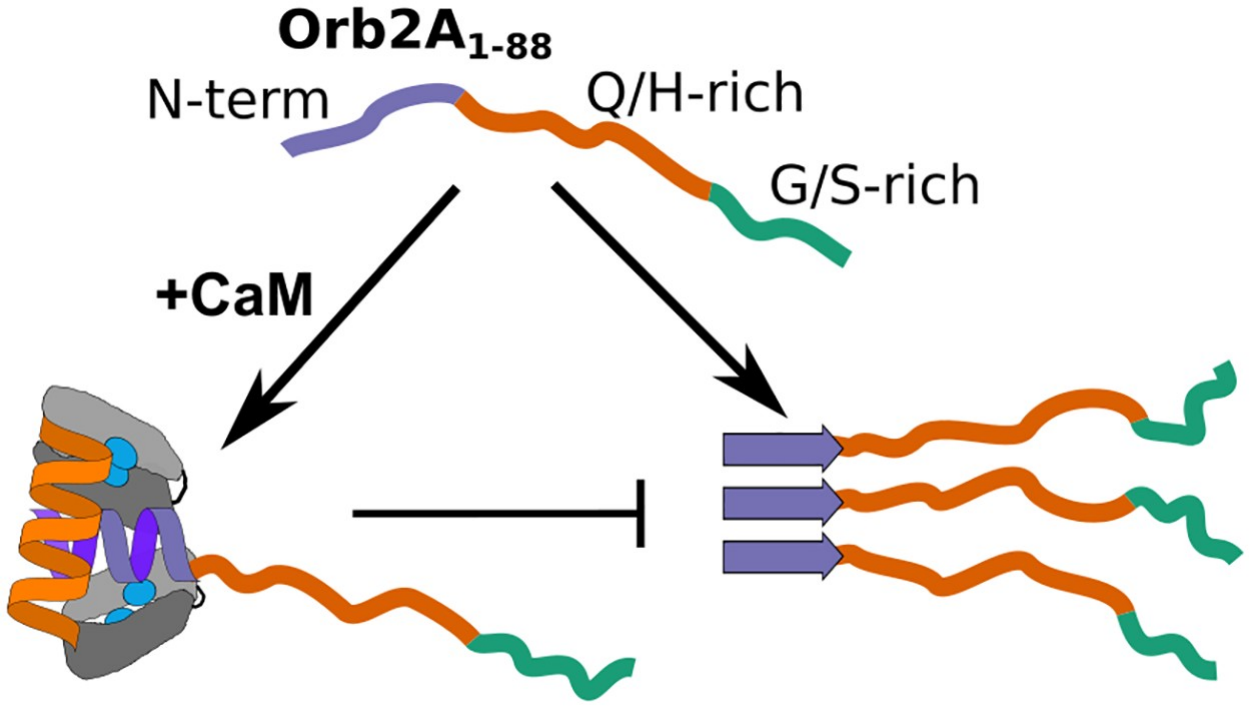
Activated CaM prevents fibril formation of Orb2A_1-88_. Addition of Ca^2+^activated CaM sequesters the N-terminal amphipathic region of Orb2A_1-88_. This interaction prevents the formation of N-terminal β-sheets necessary for cross-β fibril formation.

Our EPR, ThT fluorimetry and EM data show that CaM inhibits Orb2A_1-88_ fibril formation in the presence of Ca^2+^. This was expected because the N-terminal amphipathic domain that interacts with CaM also forms the cross-β core of Orb2A_1-88_ fibrils [8]. Previously, we showed that the binding of the N-terminal amphipathic domain to lipid membranes also inhibited fibril formation [11]. We hypothesized that binding to lipid membranes stabilized an N-terminal helix thereby not allowing the same domain to form a cross-β structure. CaM binding of Orb2A_1-88_ could create a similar situation where the N-terminus is stabilized in a helical conformation unable to interact with other Orb2A_1-88_ monomers to form a cross-β fibril (see Fig 6).

Although our data show that the CaM-Orb2A_1-88_ interaction is specific and long-lasting, the dissociation constant we determined (1.6 μM) is higher than for most CaM targets which have *K*ds in the range of 10^−7^ to 10^−11^ M [35]. However, there are several reports of similar or even higher dissociation constants in the literature [36, 37].

Calcium signaling is very important for LTM, and CaM is an integral mediator of Ca^2+^ signaling inside neurons. A key function of CaM in this context is to bind and activate the kinase CaMKII and other enzymes that are important for synaptic plasticity [12, 14–20]. Interestingly, some CPEB homologues, such as CPEB-1 in mice and CPEB in *Xenopus* oocytes, are activated when phosphorylated by CaMKII [23, 38]. Although White-Grindley and co-workers showed that phosphorylation of Orb2A induced long-term memory by increasing its half life, it is the Tob (Transducer of Erb-B2) dependent phosphorylation of Orb2 via the Lim Kinase rather than CaMKII that is responsible for this effect [39]. It is interesting to speculate that in the case of Orb2A, activation could happen by the direct interaction with CaM, thereby circumventing the extra step of phosphorylation via CaMKII.

However, we do not know if and how binding of CaM to the N-terminus specific to Orb2A can affect learning and memory in vivo. In addition, why would CaM binding to Orb2A_1-88_ prevent its fibril formation if both Ca^2+^ activated CaM and Orb2 aggregation are necessary for long-term memory? The answer to this question might come from the fact that although the N-terminal, amphipathic domain of Orb2 is important for initiating Orb2 fibril formation necessary for LTM [2, 3, 7], the fibril core of functional Orb2 fibrils is located in the Q/H-rich domain that is shared between Orb2A and B rather than the N-terminus specific to Orb2A [5]. Our previous work has shown that the N-terminus of Orb2A can form cross-β fibrils on its own in Orb2A_1-88_. In this construct, Q/H-rich fibril core of Orb2 was not observed. On the other hand, Orb2AΔRBD fibrils that were made via phase separation showed the presence of the Q/H-rich fibril core rather than the N-terminal core [6], suggesting that these cores might be mutually exclusive. Similarly, mutually exclusive fibril cores were recently reported for TDP-43 [40]. In a scenario where only either the N-terminal or the Q/H-rich core of Orb2A can exist, the binding of CaM could facilitate the formation of the latter by preventing the formation of the former.

Another solution to the apparent contradiction that CaM interaction prevents fibril formation could have something to do with the fact that Orb2 aggregation is only associated with LTP maintenance and not initiation. For example, CaMKII activation becomes independent from CaM binding when the cell switches from LTP initiation to LTP maintenance. Similarly, Orb2A aggregation could initially be inhibited by CaM binding but move forward at a later point to form Orb2 fibrils important for LTP maintenance but not initiation.

Both hypotheses are compatible with our previous proposal that the cross-β core found within the N-terminal amphipathic domain of Orb2A is potentially transient or regulatory for the more permanent Q/H-rich core found in functional Orb2 in vivo [5, 8]. More studies are needed to fully understand the molecular processes involved in LTM in general, and particularly on the regulation of Orb2A fibrillization in cells and in vivo.

## Supporting information

S1 Data Data from graphs found in the paper. Plot_Data_Orb2-CaM_interaction.
(XLSX)
